# Tamas Pataki's (2014) “Wish-fulfillment in philosophy and psychoanalysis”

**DOI:** 10.3389/fpsyg.2014.01378

**Published:** 2014-11-26

**Authors:** Andrew C. Sims

**Affiliations:** School of Humanities and Social Sciences, Faculty of Arts and Education, Deakin UniversityBurwood, VIC, Australia

**Keywords:** wish-fulfillment, intention, agency, commonsense psychology, mechanism

Wish-fulfillment is a psychoanalytic concept that applies to situations in which some agent with a frustrated desire represents the world as he would like it to be—rather than as it actually is—and in this manner pacifies the desire. This substitution by the agent of his phantasy for a veridical view of what is going on with him and the external world has been thought by psychodynamic theorists since Freud to explain a wide range of phenomena: the content of dreams, psychiatric delusion, religious practices, and so on. In this book Tamas Pataki gives a theory of wish-fulfillment which is meant to unify and explain a range of phenomena that does justice to the original explanatory scope of the concept, from neurosis and delusion to art and religion. Pataki's book shows up against the background of a tradition in analytic philosophy that is generally sympathetic to psychoanalytic ways of thinking about mindedness, and which deploys its resources in order to (i) better understand the patterns of explanation that are given in clinical practice, and the concepts that those explanations employ; and (ii) outline the necessary conditions on any such explanation—to determine what the mind must be like, if psychoanalytic explanation is cogent. One discovery that issues from this kind of analysis is that psychoanalysis constitutes an extension of ordinary psychological explanation. This was first comprehensively argued by Wollheim (1971) and his colleague Hopkins (1982), with later extension and qualifications of that argument by Gardner (1993) and Lacewing (2012).

Pataki's own treatment is remarkable in that it espouses the radical extension of agency (what he calls “intentionalism”) where past treatments—like the Bayesian story in Hopkins (2012)—have largely attempted to cash out psychoanalytic concepts in deflated (or “sub-intentional”) terms; and lately even in terms that interface with the sub-personal theories of cognitive science. Pataki is not unaware of the conceptual issues with positing unconscious centers of agency, and gives a closely argued defense of his thesis that the intentional idiom can be applied all the way down. One impressive thing about this book is the way that the author always has an eye on criticisms that might come from mainstream philosophy of cognition; that is not always something that is present in these kinds of discussions. Thus, we get a digression in the fourth chapter on the empirical plausibility of unconscious attention and unconscious intention, in which Pataki discusses recent work in the clinical neuropsychology of anosognosia for hemiplegia.

There is in fact an interesting ambivalence toward the neurological explanation of psychopathology that runs its way through the book. Pataki has no problem in showing us that he is well across the relevant literature, particularly that in which psychodynamic theory is applied in a neuroscientific context. He even cites much of this work approvingly. But he is skeptical about the reach of neuroscience when it comes to mental illness. More specifically, he claims that a neurological account of some mental illness cannot constitute a *sufficient* account of that illness; that is to say that he endorses the indispensability of the Intentional idiom. The grounds for this skepticism lie in a view about mental states according to which the facts on which their contents supervene exceed facts about the brain; and more specifically, that they supervene also on the causal histories and objects of those states. Thus, the Intentional vocabulary of mental states has autonomy from the vocabulary of neural states, and Pataki thinks that this licenses him in making a hard-and-fast distinction between neurological disorders (like Parkinson's) and mental disorders (like schizophrenia). In the latter cases, Pataki maintains, a full account of any particular disorder requires a specification in terms of what the symptoms mean for the patient.

Pataki's positive thesis is that wish-fulfillment necessitates the agent creating evidence for himself in order to elicit a particular false belief, and that this evidence-creation is performed intentionally, albeit unconsciously. Pataki gives the compelling example of the exhibitionist who exposes himself to women in order to garner the evidence that his genitals are impressive and important. The shock and outrage of his victims constitutes the evidence for this wished-for state of affairs, and enables the exhibitionist to believe that it is true. But how is this performed without falling into Sartrean paradox? Pataki claims that the right analysis of this intentional wish-fulfillment will show that wish-fulfillment involves the agent becoming engrossed in playing the role of various *personations* which have distinct constellations of beliefs and desires, and that are activated by the agent in response to certain triggers or anxieties. This activation of personations, which are often derived from representations of first caregivers, constitutes a kind of self-caring that proceeds through the gratification of the agent's frustrated wishes.

I feel that there is a possible equivocation in the book between a weak view on which the Intentional idiom is indispensable in the explanation of mental illness, and on a strong view on which it is sometimes sufficient. I also think it may be that the general mechanisms of mental illnesses are best specified in the language of cognitive neuroscience, and that this specification will not require any Intentional supplement. That may not be incompatible with the thesis that some illness-tokens are interpretable in terms of wish-fulfillment, but it may cause problems for the view that illnesses like schizophrenia will always come under a psychoanalytic description.

These concerns aside, some of the richest material in the book comes when Pataki is demonstrating the explanatory payoffs of his theory of wish-fulfillment. Indeed, one of the benefits of Pataki's account is that it can illuminate a much wider range of phenomena than is possible if wish-fulfillment is deflated to be a purely causal phenomenon. In the second half of the book Pataki goes on to apply his theory to delusion and self-deception, the psychological origins of religious belief, creative writing, and the insanity defense in jurisprudence. Indeed, it may be that the light that Pataki's theory is able to cast on a wide range of psychological and cultural phenomena leads the reader to bite the bullet and accept that agency goes much deeper than we have traditionally thought to be the case. In any case, this is a deep and interesting book, and although it is in some ways unfashionable in the context of the contemporary literature it is nonetheless quite compelling in the way it presents the case for the centrality of wish-fulfillment in human mindedness.

## Conflict of interest statement

The author declares that the research was conducted in the absence of any commercial or financial relationships that could be construed as a potential conflict of interest.
